# Short-chain fatty acids promote the effect of environmental signals on the gut microbiome and metabolome in mice

**DOI:** 10.1038/s42003-022-03468-9

**Published:** 2022-05-31

**Authors:** Francesco Marrocco, Mary Delli Carpini, Stefano Garofalo, Ottavia Giampaoli, Eleonora De Felice, Maria Amalia Di Castro, Laura Maggi, Ferdinando Scavizzi, Marcello Raspa, Federico Marini, Alberta Tomassini, Roberta Nicolosi, Carolina Cason, Flavia Trettel, Alfredo Miccheli, Valerio Iebba, Giuseppina D’Alessandro, Cristina Limatola

**Affiliations:** 1 Department of Physiology and Pharmacology, Sapienza University, Rome, Italy; 2 Center for Life Nanoscience Istituto Italiano di Tecnologia@Sapienza, Rome, Italy; 3 Department of Chemistry, Sapienza University of Rome, Rome, Italy; 4 NMR-Based Metabolomics Laboratory (NMLab), Sapienza University of Rome, Rome, Italy; 5 EMMA CNR, Monterotondo, Italy; 6 Department of Medical Sciences, University of Trieste, Trieste, Italy; 7 Department of Environmental Biology, Sapienza University of Rome, Rome, Italy; 8 IRCCS Neuromed, Pozzilli, IS Italy; 9 Department of Physiology and Pharmacology, Sapienza University, Laboratory affiliated to Istituto Pasteur Italia, Rome, Italy

**Keywords:** Neurophysiology, Microbiome, Molecular neuroscience, Metabolomics

## Abstract

Gut microorganisms and the products of their metabolism thoroughly affect host brain development, function and behavior. Since alterations of brain plasticity and cognition have been demonstrated upon motor, sensorial and social enrichment of the housing conditions, we hypothesized that gut microbiota and metabolome could be altered by environmental stimuli, providing part of the missing link among environmental signals and brain effects. In this preliminary study, metagenomic and metabolomic analyses of mice housed in different environmental conditions, standard and enriched, identify environment-specific microbial communities and metabolic profiles. We show that mice housed in an enriched environment have distinctive microbiota composition with a reduction in gut bacterial richness and biodiversity and are characterized by a metabolomic fingerprint with the increase of formate and acetate and the decrease of bile salts. We demonstrate that mice treated with a mixture of formate and acetate recapitulate some of the brain plasticity effects modulated by environmental enrichment, such as hippocampal neurogenesis, neurotrophin production, short-term plasticity and cognitive behaviors, that can be further exploited to decipher the mechanisms involved in experience-dependent brain plasticity.

## Abstract (web summary)

Mice exposed to environmental enrichment for 5 weeks display distinct microbiota composition and behavioral and metabolic profiles compared to mice exposed to a standard environment. Mice treated with a mixture of short-chain fatty acids that are produced by gut bacteria recapitulate some of the effects of this environmental enrichment.

## Introduction

Environmental enrichment (EE) represents a housing condition modified to enhance the physical and psychological welfare of experimental/captive animals in the effort to satisfy the specie-specific needs^1^. Housing enrichment is obtained providing animals with social, sensorial and motor stimuli and many evidences demonstrated beneficial effects in improving cognition, memory and motor activities in physiological conditions and in models of neurodegenerative diseases. In particular, EE-housed mice show increased neuronal survival, dendritic branching, synaptogenesis and neurogenesis in the hippocampus and in cortical regions^2–4^; further effects include decreased depressive-like behaviors and improved learning and memory^5,6^. At molecular level, the beneficial effects of EE have been often attributed to the increased expression of neurotrophins such as BDNF^7–10^, NGF^11,12^ as well as to the higher expression of synaptic proteins such as synaptophysin and PSD-95^13^. One additional molecular mediator of the environmental effects on neurogenesis comprises adiponectin^14,15^.

The gut microbiota is the community of commensal microorganisms present at the epithelial barriers of the gastro-intestinal tract. It can be shaped by genetic and epigenetic factors, being modulated by diet, drugs and lifestyle^16^. In addition to regulate the maturation and development of the immune system, recent evidence demonstrated the implication of the gut microbiota in several functions of the CNS, affecting the permeability of the blood–brain barrier^17^, the functions of glial cells^18^, and regulating complex behaviors such as mood^19^ and feeding behavior^20,21^.

Dysfunctions of the gut microbiota due to diet, metabolic diseases or drug treatment have been associated with several neurodegenerative and psychiatric diseases and with traumatic brain injury^22^. Modifications in gut microbiota composition imply modifications of microbe-derived factors such as bile salts, bacterial structural proteins, molecules associated to microbes (MAMPs; microbes associated molecular patterns), short-chain fatty acids (SCFAs), and neurotransmitters. The most abundant metabolites derived by microbial fermentation of diet fibers are the SCFAs, small molecules with one to four carbon atoms (formate, acetate, lactate, propionate, butyrate) which play local and systemic roles in shaping immunity, maintaining gut and blood–brain barrier integrity, and promoting microglia maturation and function and preventing neuroinflammation^23^. Furthermore, altered levels of SCFAs have been described in neurodegenerative diseases, such as Parkinson’s and Alzheimer’s diseases^24,25^ and in stroke^26^.

Recent evidence describes that housing animals in EE and prescribing to humans the translatable lifestyles result in improved cancer immunosurveillance and in a better patient response to cancer immunotherapies^27^. In this study we hypothesized that the housing conditions affect the population of gut resident microbes and their metabolic products, and we performed metagenomic and metabolomic analyses of feces produced by mice housed in standard environment (SE) or EE. Among the other variations, we describe that the mice housed in an EE have a reduced amount of bile salts and an increased abundance of formate and acetate in their fecal water. Feeding mice housed in SE with a mixture of these SCFAs mimics some of the behavioral and molecular effects of environmental enrichment, such as reduced anxiety, increased memory retention, neurogenesis and expression of neurotrophins such as *bdnf* and *vegf-a* in the hippocampal region. We also observed a reduced paired-pulse ratio (PPR) in mice treated with SCFA, suggesting an enhanced hippocampal probability of glutamate release. These data suggest that gut microbes rapidly adapt to environmental signals and to changes in the host lifestyle, which deeply influence the microbe network population. Microbial products, namely formate and acetate, could represent molecular effectors of the enriched environment in the CNS.

## Results

### Housing mice in EE modifies gut microbiota composition

**Fig. 1:**
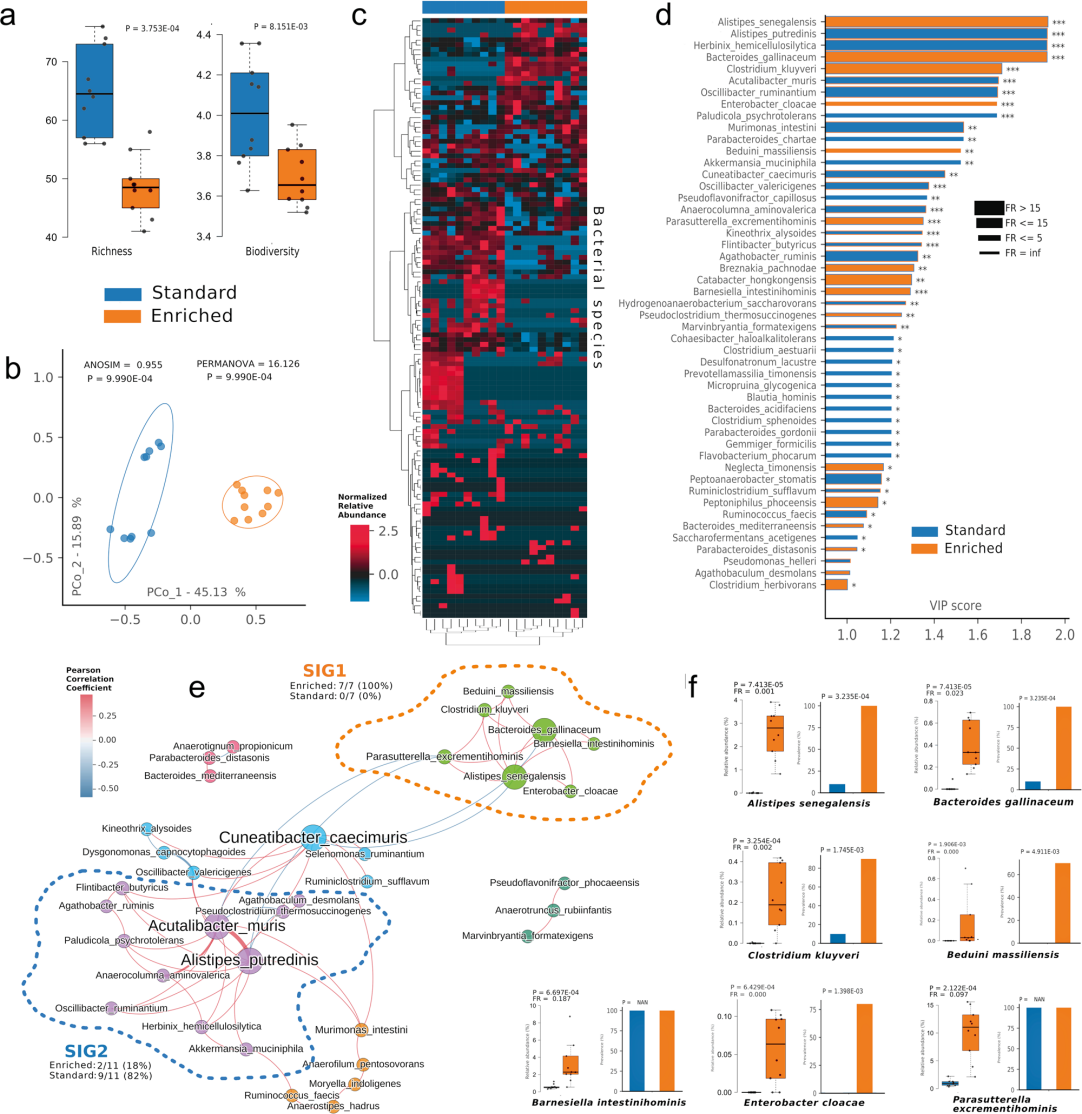
Fecal microbiota composition in EE and SE mice. Alfa-diversity (**a**) shows differences in both richness (number of bacterial species) and biodiversity (Shannon metric) among standard (SE, blue, *n* = 10 biologically independent samples) and enriched mice (EE, orange, *n* = 10 biologically independent samples). Beta-diversity (**b**) and Hierarchical Clusterization Analysis (HCA, **c**), following the Bray-Curtis dissimilarity distance algorithm, both show significant separation of SE and EE. Variable Importance Plot (VIP, **d**) shows: (i) discriminant species after PLS-DA in descending order of VIP score (bar length); (ii) the highest relative abundance depending on the cohort (central bar color) and the lowest one (edge bar color); (iii) fold ratio (FR) of the highest vs the lowest relative abundance (bar thickness); (iv) significant difference after Mann–Whitney *U* test (non-FDR, *P ≤ 0.05, **P ≤ 0.01; ***P ≤ 0.001). Network analysis (**e**) shows communities of bacterial species (namely species-interacting groups, SIGs) and their positive (red Pearson coefficient) or negative (blue Pearson coefficient) relative abundances correlation. Nodes are colored according to the cohort harboring the higher relative abundance for a definite species, and node name size is directly proportional to the “keystonness” (importance of a species within the overall network). Edge thickness is inversely proportional to the Pearson P value after 10% Benjamini-Hochberg two-stages FDR, and it is colored according to positive (red) or negative (blue) Pearson coefficient. For each SIG are reported percentages of EE- and SE-related species. Pairwise analysis (**f**) of 7 selected species (belonging to EE-related SIG1) depicts significant differences in terms of relative abundance and prevalence when matched with SE cohort. In each sub-graph are reported the *P* value (from Mann–Whitney *U* test) and the fold ratio (FR) among SE and EE cohorts. The boxplots show the minimum, 25th percentile, median, 75th percentile, and maximum values. Error bars = SEM.

To determine if stimuli from the external environment could influence the composition of gut microbiota, ten mice were housed in SE or EE, as described in the methods, for five weeks and the alpha and beta-diversity in gut microbiome and the composition of the fecal microbial community were determined by 16S rRNA sequencing. As shown in Fig. 1a, fecal microbiota of mice housed in EE shows a reduction in bacterial richness and diversity indexes compared to mice housed in SE, as reflected by the significant reduction in richness (*p* = 0.00037) and Shannon indexes (*p* = 0.0081). An unsupervised ordination plot (Principal Coordinate Analysis, PCoA, Fig. 1b) evidenced a clear separation among EE and SE cohorts (PERMANOVA *p* = 0.00099, ANOSIM *p* = 0.00099), revealing differences in the two microbiota compositions. Due to the relatively low number of mice used for this comparison, further experiments with higher number of animals will be necessary to consolidate these results in a more robust statistical analysis. To exclude the possibility that the differences of microbiota composition between the groups are due to a cage effect because of the coprophagic behavior of mice, we also performed a longitudinal analysis of alpha and beta diversity. As shown in Supplementary Fig. 2a, b, the effect of housing conditions predominates over the cage effect and confirms that the reported differences were not due to initial interindividual differences. We speculate that the differences observed among basal and SE group are likely due to different diets at the beginning (maternal milk) and at the end (regular chow) of the 5 weeks period, but further experiments specifically addressing this point will be necessary to demonstrate it. A further confirmation of the good separation between the SE and EE housed groups derives from the unsupervised Hierarchical Clusterization Analysis (HCA, Fig. 1c), which evidences two definite clusters (Fisher’s exact test *p* < 0.0001). To possibly identify bacterial species distinctive for EE or SE cohorts, Partial Least Squares Discriminant Analysis (PLS-DA) model and the VIP score were implemented (Fig. 1d). At least fifteen species were distinctive for the EE group, and seven were more significantly enriched in EE, as shown in the pairwise analysis (Fig. 1f and Supplementary Data 1): *Alistipes senegalensis*; *Bacteroides gallinaceum*, *Clostridium kluyveri*, *Enterobacter cloacae*, *Beduini massiliensis*, *Parasutterella excrementihominis*, *Barnesiella intestinihominis*. Interestingly, when we applied a network analysis approach to study the putative formation of bacterial consortia named species-interacting-group (SIG), these seven species were all included within a single community named SIG1 (Fig. 1e), which was significantly opposed to SIG2, a community mainly inhabited by species related to SE (Fisher’s exact test *p* = 0.0023). Noteworthy, the two *Alistipes* species being distinctive for EE and SE, respectively *A. senegalensis* and *A. putredinis*, fell within the two opposite SIGs. When compared to the basal microbiome composition (time 0), *A. senegalensis, B. gallinaceum, B. massiliensis*, and *P. excrementihominis* increased in EE condition, suggesting an environment-induced species-specific variation (Supplementary Fig. 2c). Altogether, these data indicate that EE induces changes in gut microbiota composition, allowing the growth of definite bacterial species which positively clustered together.

### Exposure of mice to EE changes gut microbiota metabolome

Forty-three molecules were identified, and thirty-seven metabolites were quantified by 1D ^1^H-NMR analyses and considered in multivariate analysis for eight SE and ten EE mice (Supplementary Data 2). A representative fecal water ^1^H-NMR spectrum is reported in Supplementary Fig. 3.

The resonance of C-18 protons of bile salts moieties was not univocally assigned to a specific molecule, since the characterization is very challenging due to the presence of overlapped peaks (Supplementary Fig. 4). In fact, each bile salt corresponds to several molecules which were identified on the basis of the chemical shifts, as previously reported^28,29^. In detail, bile salt 01 resonance is made up by C18 protons of glycochenodeoxycholate, taurochenodeoxycholate, γ-muricholate, chenodeoxycholate, taurocholate; bile salt 02 by β-muricholate, ω-muricholate, taurodeoxycholate, cholate, glycocholate; bile salt 03 by deoxycholate, glycodeoxycholate, and tauroursodeoxycholate.

**Fig. 2:**
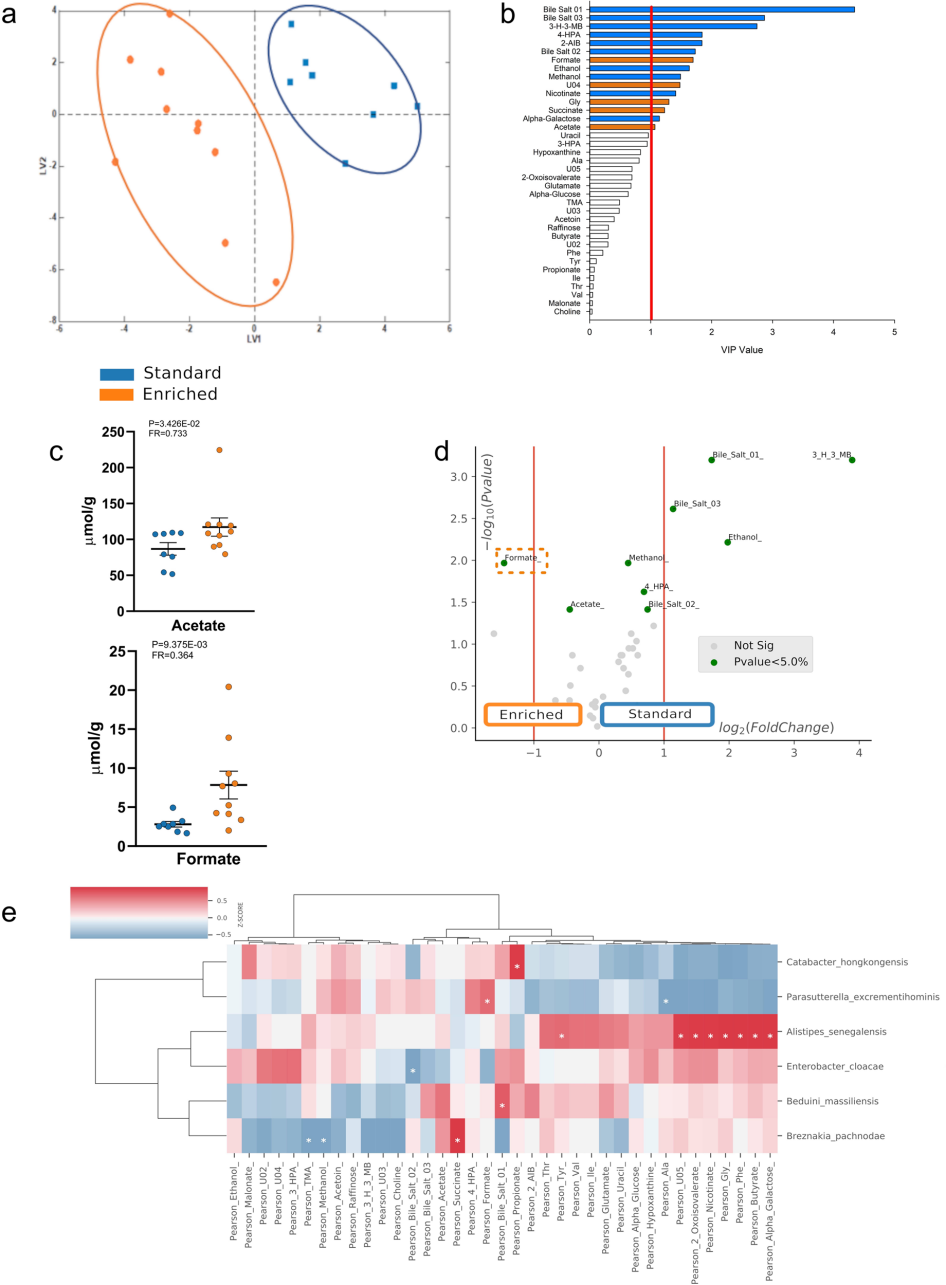
Comparison between stool metabolome of mice housed in EE and SE. **a** Latent Variables Score plot by double cross validation PLS-DA. Orange: EE, blue: SE; **b** VIP values are reported in horizontal histogram, values of VIP above 1 are statistically significant. **c** comparison of fecal levels of formate and acetate between EE and SE mice. **d** Volcano Plot showing the changes of fecal metabolites in different housing conditions. **e** cross-correlation heatmap within EE population and metabolites. Each unit describes Pearson correlation value as z-score. 3-H-3-MB 3-hydroxy-3-methylbutyrate, 4-HPA 4-hydroxyphenylacetate, 2-AIB 2-aminoisobutyrate, Gly glycine, 3-HPA 3-hydroxyphenylacetate, Ala alanine, TMA trimethylamine, Phe phenylalanine, Tyr tyrosine, Ile isoleucine, Thr threonine, Val valine, U unassigned resonance. The boxplots show the minimum, 25th percentile, median, 75th percentile, and maximum values. Error bars = SEM.

In accordance with the metagenomic analysis, fecal NMR-based metabolomics of mice housed in EE and in SE revealed different profiles as shown in the PLS score plot (LV1 versus LV2) in Fig. 2a. Specific metabolites characterize both EE and SE conditions with high classification scores (EE: 78.8 ± 8.9%; SE: 98.2 ± 4.3% SE *n* = 8 biologically independent samples; EE *n* = 10 biologically independent samples) as shown by double crossed-validated PLS-DA analysis using VIP scoring. In fact, as shown in Fig. 2b, the abundances of fecal metabolites changed in the EE-housed mice with respect to SE mice. Among the identified metabolites, formate, glycine, succinate, and acetate significantly increase in EE. On the contrary, bile salt 01, bile salt 03, 3-H-3-MB, 4-HPA, 2-AIB, bile salt 02, ethanol, methanol, nicotinate, and alpha-galactose are less abundant in EE (Supplementary Data 3).

Two molecules, identified as key metabolites for EE were SCFAs, among the most abundant microbial-derived factors. Figure 2c shows the quantification of fecal acetate and formate: in EE-housed mice, acetate level was 1.35 folds higher than in SE (SE: 86.80 ± 8.78 μmol/g stool; EE: 117.15 ± 12.7 μmol/g stool; *P* = 0.03426) (SE *n* = 8 biologically independent samples, EE *n* = 10 biologically independent samples) while formate increased 2.81 folds (SE: 2.79 ± 0.35 μmol/g stool; EE: 7.84 ± 1.7735 μmol/g stool *P* = 0.009375).

Interestingly, among the EE group, formate obtained the highest VIP score (Fig. 2b), and the most significant change in the volcano plot (Fig. 2d), thus representing a key metabolite in discriminating the EE group.

Cross-correlating the two datasets of metabolites and bacterial species, we found that six species characterizing the EE housing condition (Fig. 1d), and mainly belonging to SIG1 community (Fig. 1e), were separated in two clusters: (1) *Parasutterella excrementihominis* and *Catabacter hongkongensis*, and (2) *Alistipes senegalensis, Enterobacter cloacae, Beduini massiliensis* and *Breznakia pachnodae* (Fig. 2e). Even if *P. excrementihominis* is the only specie that significantly correlates with formate levels (*ρ* = 0.67, *p* = 0.049), other bacterial species were positively related to metabolites involved in brain plasticity (*Alistipes senegalensis* vs glycine *ρ* = 0.94, *p* = 1.2×10-4; *Breznakia pachnodae* vs succinate *ρ* = 0.96, *p* = 3.1×10-5; *Catabacter hongkongensis* vs propionate *ρ* = 0.90, *p* = 1.1 × 10^−3^) (Supplementary Fig. 5 and Supplementary Data 4). Taken together these data identify SCFAs as potential mediators of at least some of the effects induced by housing animals in an EE^2^.

### SCFA administration affects mouse behavior, hippocampal neurogenesis, and neurotrophin expression

To verify the hypothesis that EE-induced microbiome modification could be involved in EE-mediated effects on neuronal plasticity^30^, we treated mice with a mixture of selected SCFAs which were increased by EE housing, such as sodium formate and acetate (1:0.45 ratio). SCFAs, dissolved in drinking water, were given to mice for 5 weeks, and animals were then analyzed for behavior modification, hippocampal neurogenesis, neurotrophin expression, short- and long- term plasticity.

**Fig. 3:**
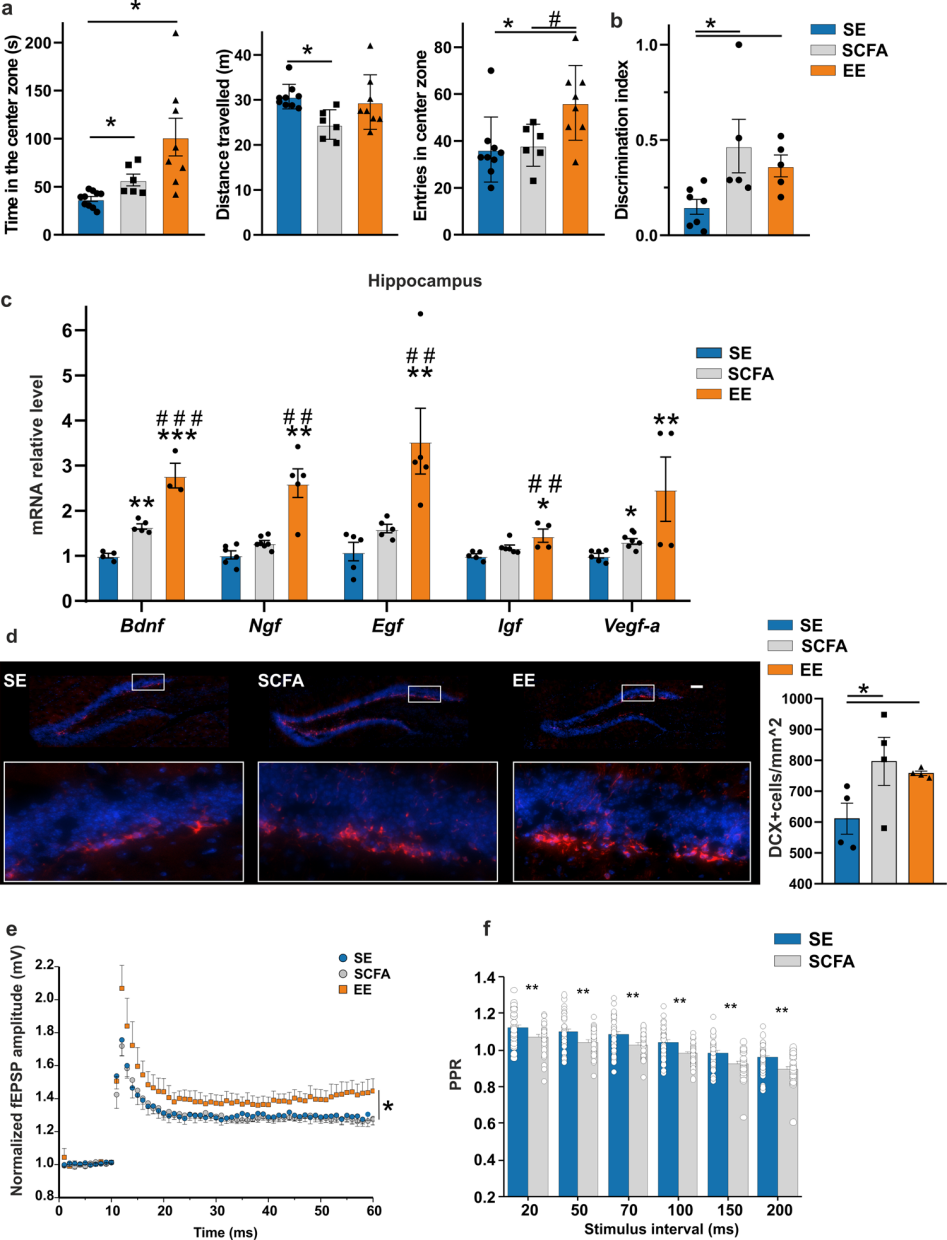
Formate and acetate administration induces brain plasticity in EE-housed mice. **a** Open field test for mice SE, treated with SCFA (150 mM sodium formate and 62.5 mM sodium acetate) or exposed to EE environment for 5 weeks: left panel, time (in seconds) spent in the center zone; central panel, distance traveled (in meters) during the test; right panel, number of entries in the center zone (*p-value < 0.05 vs SE; #*p*-value < 0.05 vs SCFA. Data are the mean ± S.E.M.). **b** Novel object recognition test for SE, SCFA and EE mice. The discrimination index was calculated as the amount of time spent exploring the novel object relative to the familiar object, divided by the total amount of time exploring both objects. SCFA and EE mice performed better than controls (*p-value < 0.05 vs SE. Data are the mean ± S.E.M.) **c** RT-PCR of hippocampal neurotrophins (*Bdnf, Ngf, Egf, Igf, Vegf-a*) in SE, SCFA- or EE treated mice (***p < 0.001; **p < 0.01; *p < 0.05 vs SE; ###*p* < 0.001; ##*p* < 0.01 vs SCFA. Data are the mean ± S.E.M.). **d** Representative 20x magnification of mouse hippocampal dentate gyrus (DG) coronal sections stained for DCX (red) and Hoechst (blue) of SE, SCFA- and EE treated mice (scale bar, 100 μm). The highlighted squares illustrate the DG regions digitally amplified where DCX + cells are shown. Right, bar graphs showing the number of DCX + cells for mm^2^ in the DG of the three groups (*p-value < 0.05 vs SE. Data are the mean ±  S.E.M.). **e** Field excitatory postsynaptic potentials (fEPSPs) recorded from the CA1 region of hippocampus of SE (blue), SCFA (gray), or EE (orange) mice following Schaffer Collateral high frequency stimulation (100 Hz, of 1 s duration, arrow). Points represent mean (± SEM) of normalized fEPSP amplitude slopes evoked every 20 s (*p < 0.05). **f** Paired-pulse ratio (PPR) for 20-50-70-100-150-200 ms inter-stimulus intervals. Bar histograms represent averaged values for SE and SCFA (**p < 0.01).

We first tested mouse behavior in the open field test, and measured the time spent by the animals in the center of the arena and the distance traveled during the observation time (10 min), parameters correlated with an anxiety-like behavior^31^. Data in Fig. 3a show that in the open field test, SCFA-treated mice spent more time in the center of the arena (SE = 35.7 ± 1.9 s *n* = 9 biologically independent animals, SCFA = 57.0 ± 7.5 s, *n* = 6 biologically independent animals *p* < 0.05 vs SE; EE = 101.7 ± 19.5 s *n* = 8 biologically independent animals by ANOVA Dunn’s post-test), a behavior similar to what observed in EE-housed mice. In addition, SCFA-treated mice traveled significantly less compared to control mice (SE = 30.8 ± 2.2 meters *n* = 9 biologically independent animals, SCFA = 24.5 ± 1.3 meters *n* = 6 biologically independent animals *p* < 0.050 vs SE, EE = 29.5 ± 2.1 meters *n* = 8 biologically independent animals by by ANOVA Dunn’s post-test), while no effects were observed in the number of entries in the center zone in 10 min (SE = 39.0 ± 4.4 entries *n* = 9 biologically independent animals, SCFA = 38.1 ± 3.6 entries *n* = 6 biologically independent animals, EE = 56.2 ± 5.6 entries *n* = 8 biologically independent animals *p* < 0.05 vs SE and *p* < 0.05 vs SCFA by ANOVA Dunn’s post-test) (Fig. 3a). These data indicate a reduced anxiety-like behavior of SCFA-treated mice in comparison to SE mice^32^, similarly to what observed in EE-housed mice.

To investigate the effect of SCFA treatment on short-term memory, mice were analyzed in the novel object recognition test (NORT). Figure 3b shows that SCFA-treated mice spent more time exploring novel objects, as shown by the higher discrimination index, similar to EE-housed animals (SE = 0.16 ± 0.04, *n* = 7 biologically independent animals, SCFA = 0.46 ± 0.14, *n* = 5 biologically independent animals *p* < 0.05 vs SE, EE = 0.36 ± 0.05, *n* = 5 biologically independent animals *p* < 0.05 vs SE by ANOVA Dunn’s post-test) indicating a better recognition memory than vehicle-treated SE housed mice, and suggesting that learning is enhanced by SCFA treatment and EE.

We then wanted to verify the hypothesis that, similarly to EE, the effects of SCFAs on short-term memory could be related to increased hippocampal neurotrophin expression and to neurogenesis. RT-qPCR analysis of the hippocampal regions demonstrates an increased expression of *Bdnf* (SE = 1.00 ± 0.05 *n* = 5 biologically independent samples, SCFA = 1.65 ± 0.06 *n* = 5 biologically independent samples *p* < 0.001 vs SE, EE = 2.78 ± 0.27 *n* = 3 biologically independent samples *p* < 0.001 vs SE and *p* = 0.004 vs SCFA by ANOVA Student-Neuman-Keuls post-test), *Vegf-a* (SE = 1.00 ± 0.05 *n* = 6 biologically independent samples, SCFA = 1.38 ± 0.04 *n* = 9 biologically independent samples *p* < 0.05 vs SE, EE = 2.47 ± 0.71 *n* = 4 biologically independent samples *p* < 0.05 vs SE by ANOVA Dunn’s post-test) both in SCFA-treated and in EE-housed mice while other neurotrophins found to be increased in EE mice did not significantly change upon SCFA treatment (Fig. 3c). Immunofluorescence analysis of the hippocampal region shows a higher number of neuronal precursors, identified as doublecortin (DCX) positive cells into the dentate gyrus (DG) of SCFA-treated and in EE-housed mice compared to controls (SE = 611.0 ± 50.8 cells/mm^2^ *n* = 4 biologically independent samples, SCFA = 796.2 ± 78.2 cells/mm^2^ *n* = 4 biologically independent samples *p* < 0.050 vs SE, EE = 758.0 ± 7.3 cells/mm^2^ *n* = 4 biologically independent samples *p* < 0.05 vs SE by ANOVA Student-Neuman-Keuls post-test), as shown in Fig. 3d^33^.

To evaluate whether the long-term potentiation (LTP) of excitatory neurotransmission in the CA1 region of the hippocampus, known to be increased by EE, could be affected by the gut metabolites, we recorded the LTP induced by 100 Hz stimulation of Schaffer Collateral inputs comparing control with EE and SCFA-treated mice. As shown in Fig. 3e, mice receiving SCFAs have similar LTP amplitude in the CA1 synapses (1.28 ± 0.01, n/N = 10 biologically independent samples/4 biologically independent animals, *p* = 0.99 vs SE by ANOVA, Dunnett’s post-hoc test) compared to SE (1.28 ± 0.03, n/N = 11 biologically independent samples/4 biologically independent animals), whereas EE mice have significantly increased LTP amplitude (1.41 ± 0.05, n/N = 6 biologically independent samples/2 biologically independent animals, *p* = 0.046 vs SE by ANOVA, Dunnett’s post-hoc test), as previously reported^34^. In addition, we measured a form of short-term plasticity associated with neurotransmitter release probability by recording CA1 paired-pulse ratio (PPR) at different inter-stimulus intervals (20-200 ms). We found that at every interval analyzed, the PPR was reduced in SCFA-treated mice (n/N = 35 biologically independent samples /6 biologically independent animals, *p* < 0.01 vs SE by Two-way RM ANOVA, Fisher LSD method) compared to SE (n/N = 31 biologically independent samples /5 biologically independent animals), as shown in Fig. 3f. These data suggest that SCFA treatment increases the probability of glutamate release but has no effect on CA1 long-term plasticity.

To evaluate the effect of five-weeks SCFA treatment on the gut microbiome composition and on metabolome, we analyzed the baseline (T0 group) and the endpoint stools of SCFA-treated and untreated SE mice. As shown in Supplementary Fig. 6, the microbiome composition of SCFA mice showed a significantly reduced specie richness compared to T0, similar to EE mice, while no differences in the biodiversity were observed among the three groups (panel a). An unsupervised ordination plot (Principal Coordinate Analysis, PcoA, panel b) evidenced a clear separation between T0 and both SCFA and SE cohorts (PERMANOVA *p* = 0.00099, ANOSIM *p* = 0.00099 *n* = 10 biologically independent samples), revealing differences in the microbiota compositions. However, SCFA mice segregated from T0 group better than SE mice, suggesting that the effect of the treatment had a more pronounced effect than time. Further, among the species significantly changed in the volcano plot for SCFA treated vs SE mice (panel c), we found *Beduini massiliensis* one out of the six EE species significantly correlated to EE metabolites (Fig. 2e).

**Fig. 4:**
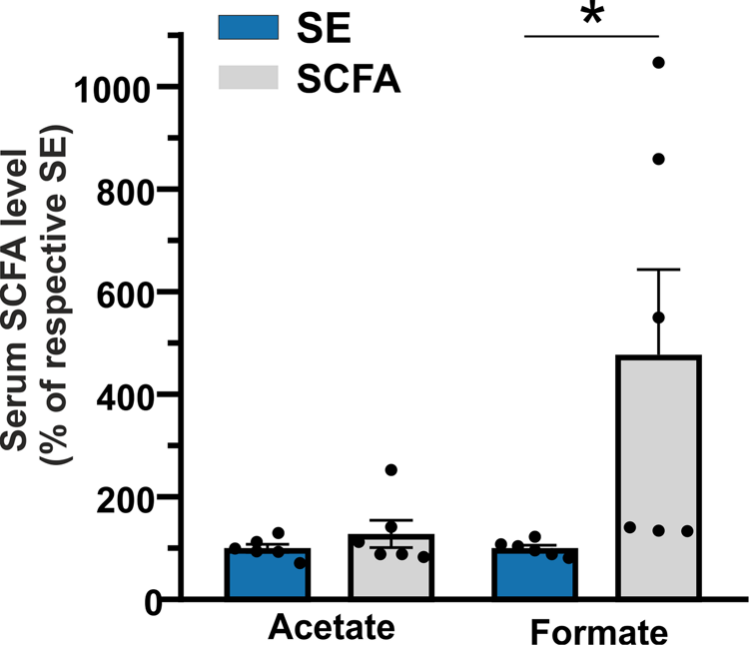
Formate and acetate serum levels in SCFA-treated mice. Serum levels of acetate and formate in mice following five-weeks SCFA treatment. Levels of SCFA in SE mice were taken as 100%. These were as follows: SE acetate 2.42 ± 0.39 mol/mol creatine and SE formate 0.18 ± 0.02 mol/mol creatine. Results are shown as percentage (SCFA, gray, acetate *n* = 6, *p* = 0.170; formate *n* = 6, *p = 0.023 vs SE by Student-t test) of respective SE mice (blue). Values are given as mean ± SEM of two independent experiments.

In parallel, we performed a PCA (principal component analysis) on NMR metabolomics variables of T0, SE, and SCFA mice. As highlighted in the PCA scores plot (Supplementary Fig. 7a), there is not a clear separation among the T0, SE, and SCFA groups. However, when we applied multivariate statistical analysis to the NMR data matrix by comparing SE and SCFA treated mice, PCA showed an apparent separation between the treated and not treated samples along the PC2 (20% of the total explained variance, Supplementary Fig. 7b). In accordance with the data described above, we did not expect many significant changes in the fecal microbiome of SCFA mice, as the absorption of acetate and formate mainly occurs in intestinal regions different from the large intestine. However, to demonstrate that orally administered SCFA could reach the blood stream and potentially directly affect brain functions, whole sera analysis of SCFA-treated mice by targeted NMR was performed. As shown in Fig. 4, levels of formate and acetate are increased in serum of SCFA-treated mice (acetate: SE 100.0 ± 8.0 *n* = 6 samples of twelve samples randomly pooled two by two; SCFA 128.0 ± 26.0 *n* = 6 samples of twelve samples randomly pooled two by two *p* = 0.170 vs SE) but only formate (SE 100.0 ± 6.0 *n* = 6 samples of twelve samples randomly pooled two by two; SCFA 477.8 ± 160.5 *n* = 6 samples of 12 samples randomly pooled two by two *p* = 0.023 vs SE) significantly changed.

## Discussion

The study of gut-brain interaction recently emerged as a new field of research and many evidences demonstrated a plethora of direct and bidirectional communication mechanisms among the two systems, and the key role played by immune cells^18^.

Gut microbiota diversity can be modified by diet composition and antibiotic treatment as well as by aging and neuropathologies^35,36^. We here demonstrated that multiple environmental signals, such as those activated by housing mice in an EE, modify the microbe population of the gut, with a sharp microbial community segregation induced by different housing conditions that highlights the powerful effect of the environment on gut microbiome.

In particular, in EE-housed mice, we observe a specific increase of species such as *Catabacter hongkongensis*, *Parasutterella excrementihominis*, *Alistipes senegalensis*, *Enterobacter cloacae*, *Beduini massiliensis*, and *Breznakia pachnodae*. An inverse variation was observed in EE between *A. senegalensis* and *A. putredinis*. *Alistipes* is a sub-branch genus of the *Bacteroidetes* phylum and, so far, it has been associated both to healthy phenotypes and pathological states^37^. The observed EE effects on microbiota composition are in line with the findings showing that social interaction can change gut microbiota through mechanisms that involve the HPA- sympathetic- enteric nervous system axis, and hormonal secretion^38^. Further, it has been found that the voluntary physical exercise can shape intestinal microbiota modifying gut inflammatory state^39^.

In accordance with the alteration of gut microbes, we demonstrate that EE modifies the panel of metabolites excreted with the feces in mice, with an enrichment in SCFAs, Gly and succinate and a decrease in ethanol, methanol and bile salts. The altered levels of formate, methanol and acetate are consistent with an induced variation of gut microbiota methane metabolism (according to the Kyoto Encyclopedia of Genes and Genomes database:^40^ Kegg pathway: M00680), in particular of methane oxidation, a methanotrophic pathway coherent with the decrease in methanol and formate increase [Kegg pathway: M00174]; Acetyl-CoA pathway (Kegg: M00422) associated to phosphate acetyltransferase-acetate kinase pathway [Kegg: M00579], as well as reductive Acetyl-CoA pathway known also as Wood-Ljungdahl pathway (WLP) [Kegg: M00377]. Through this last pathway, bacteria utilize molecular H_2,_ as the source of reducing equivalents, to reduce CO_2_ to acetate and producing energy. H_2_ generation and oxidation coupled to CO_2_ reduction to methane or acetate maintains the gut ecosystem structure. Recently, it was suggested that the reductive acetyl-CoA pathway could be coupled to hydroxyl group oxidation of biliary salts altering their hydrophobic/hydrophilic balance^41^. Our NMR analysis cannot disentangle modifications in specific bile salts; therefore, we cannot confirm this hypothesis on the basis of the co-variations among the decrease of biliary salt levels, and the increase of acetate and formate observed in EE mice.

Formate accumulation in the feces of EE-housed mice positively correlates with *Parasutterella excrementihominis*, suggesting the importance of this specie, in the modified population network, to the production of this SCFA.

In the present study, with the aim to identify possible roles of microbe-derived metabolites as mediators of the effects of the EE on learning and memory abilities, anxiety behavior, hippocampal neurogenesis and plasticity and neurotrophin production, we focused our attention on the increase of SCFAs, for their ability to affects microglial neuroimmune activities^23^. The identification of the inter-system communication molecules is of paramount interest to the knowledge of the modulation of brain and behavior by microbiota and of microbiota composition by brain activity. It has been demonstrated that periods of developmental changes in the brain parallel those of gut microbiota and a current hypothesis claims the presence of “critical windows” in the development of a functional gut microbiota-brain axis^42^.

Our observations that the EE-modified microbiota increases specific SCFAs in the feces and that the administration of a mixture of formate and acetate salts with the diet can reproduce some of the processes of neuronal plasticity induced by EE, strongly support the hypothesis that these SCFAs could play relevant role in mediating EE effects. At present, very little is known about the involvement of microbiome components in the modulation of brain plasticity. It has been recently reported that dietary supplementation with *Lactobacillus* and *Bifidobacteria* or a combination of *Actinobacter* and *Bacteroidetes* affects hippocampal plasticity^43,44^. Besides this evidence, few data are available on the role of specific microbiota metabolites on brain plasticity processes. Recently, it has been shown the sex- dependent impact of the gut microbiome on hippocampal LTP specifically in male germ-free mice^45^. Here we report that mice treatment with acetate and formate increased memory in the NOR task, supporting the hypothesis that these metabolites are actively involved in the regulation of learning related processes. Recent evidence shows that depletion of acetate-producing bacteria from the gut microbiota facilitates cognitive impairment in diabetic mice^46^. Nevertheless, we find that SCFA treatment did not affect the amplitude of hippocampal LTP: these data are compatible with the knowledge of a nonlinear relationship between LTP magnitude and memory performance^47,48^ and that different brain structures are relevant for the NOR task, such as the perirhinal cortex^49^.

Our results that SCFA treatment affects mice behavior in the open field test suggests a reduced level of anxiety, a behavior that, in addition to the hippocampus, strongly depend on the amygdala^50^. The reduced anxiety-like behavior could also contribute to the improvement observed in the NOR task. Accordingly, the oral supplementation of the classical SCFA (acetate, propionate and butyrate) in mice alleviated psychosocial stress-induced alterations and promoted behavioral test-specific antidepressant and anxiolytic effects in wt mice^51^

We report that SCFA treatment increases *Bdnf* levels in the hippocampal region. It was previously shown that oral antibiotic administration to pathogen-free mice transiently alters the composition of the microbiota, increases the exploratory behavior and the hippocampal expression of BDNF.^52^ In the hippocampus, BDNF enhances glutamate release and increases the frequency of mEPSCs^53,54^. Interestingly, we found that formate and acetate treatment reduced hippocampal short-term plasticity, indicating an increase in the release probability of glutamate at Schaffer collateral synapses. We can speculate that the mechanism underling the decrease of PPR is related to the hippocampal BDNF increase^55,56^ induced by SCFA treatment. To the best of our knowledge, this is the first evidence that oral administration of formate and acetate can affect brain neuronal transmission, and in particular, the probability of hippocampal glutamate release.

The increased expression level of *Bdnf*, together with *Vegf*-a in the hippocampus of SCFA-treated mice could be related to the enhanced neurogenesis observed in the dentate gyrus, as reported in mice exposed to EE^57,58^, supporting the hypothesis of a role of SCFAs in mediating EE-driven brain plasticity in mice.

In our study, the abundance of *A. senegalensis* in EE-housed mice was also correlated with glycine levels. Glycine has several central and peripheral activities as neurotransmitter and immunomodulatory agent^59^ and therefore we cannot exclude that some of the neurological effects induced by housing mice in EE may be related to the increase of microbial-derived glycine. Importantly, in SCFA-treated mice, serum measurements of acetate and formate show only a significant increase of formate, suggesting a different metabolic rate with possible implications in mediating the observed effects. Scant information is available on the role of formate in the gut-brain axis, this study is the first to the best of our knowledge showing the possible role of this one carbon SCFA in brain neuromodulation. The formate mainly derives from microbial methane metabolism and it is a potent regulator of purine, pyrimidine and energy metabolism of animal organism^60^. The link between microbiome composition and SCFA production in vivo are not directly predictable by the well characterized data on in vitro fermentation. However, the stool metabolic profile of EE-housed mice is more representative of a whole microbial system linked to complex interactions among the species and species/host and intestinal environment. Therefore, it is more important to define the activity of the whole microbial community than to identify the changes in a single species, investigating the gut/brain axis, in agreement with previous hypotheses^61^.

Among the questions left open by our study are the mechanisms linking gut metabolites to brain plasticity. Gut SCFAs leave the gut lumen through the portal vein; however, a concentration gradient of different SCFAs has been reported from the lumen to the periphery with the uptake of butyrate at the epithelium, propionate at the liver and acetate in the peripheral tissues^62^. SCFAs act on the target cells through multiple mechanisms including histone acetylation and methylation^63,64^, G-protein coupled receptors (GPCRs)^65^, the free fatty acid receptor 2 and 3 (FFAR 2/3), which are expressed on a wide range of cell types, including enteroendocrine and immune cells^66–68^. It has been demonstrated that classical SCFA treatment reverted immature microglia phenotype^18^, and in particular, microbiome-derived acetate is a critical driver of microglia maturation and regulator of the homeostatic metabolic state in germ-free mice^69^, and modulated microglia phenotype in a receptor-independent manner^70^. Considering the ability of microglial cells to modulate synaptic plasticity processes^71^ and the lack of FFARs expression in the brain under normal conditions^18^, we speculate that formate and acetate produced in the gut upon EE housing could indirectly modulate microglia activity with effects on synaptic plasticity. The effects of EE on brain plasticity are driven by several mechanisms in addition to microbiota^14,72^, thus our data showing a significant microbiome modification, with significant alteration of SCFA levels, indicate that these metabolites could represent one of the mediators of EE on the brain.

Although our study identifies distinct microbiota cohorts in SE and EE-housed mice, these results must be considered as preliminary, due to the relatively low number of mice used for this comparison and further experiments with higher number of animals will be necessary to consolidate these results in a more robust statistical analysis. Further, we cannot completely rule out that the observed differences among SE and EE housed animals could be due to a cage effect, and further experiments specifically addressing this point will be necessary to demonstrate it. However, we believe that as a whole the data reported in this paper open an unexplored route for the knowledge of the reciprocal influence of brain and gut microbial community.

## Methods

### Mice and environmental enrichment protocol

Experiments described in the present work were approved by the Italian Ministry of Health (authorization no. 775/2020-PR) in accordance with the guidelines on the ethical use of animals from the European Community Council Directive of 22 September 2010 (2010/63/EU), and from the Italian D.Lgs 26/2014. Male C57BL6/N mice, 3 or 4-weeks old, were obtained from the animal facility of the Physiology and Pharmacology department of Sapienza University. Only male mice were used to avoid possible sex-specific variance of microbiota, as previously shown^73^. The microbiological status of the animals and facility were continuously monitored. All mice were housed under a 12 h light/dark cycle in standard cages with autoclaved drinking water and sterilized standard chow ad libitum. For the housing in an EE, at least 10 3-week-old mice were present in a large cage (36 cm ×  54 cm × 19 cm) with 2 running wheels, long tubes, houses, nesting materials and small plastic objects of different shapes and colors for 5 weeks. The enrichment tool set was changed twice a week and all the objects were washed with soap and sanified with a 70% ethanol solution. For the housing in standard environment (SE), two or three age-matched mice were housed in standard cages (30 cm × 16 cm × 11 cm) with only nesting material for five weeks. In both experimental groups the bedding materials were changed once a week (see Supplementary Fig. 1).

### Stool collection and processing

At the beginning and at the end of the five weeks housing in enriched or standard conditions, or after SCFA treatment, fresh stools were aseptically collected, frozen and stored for metagenomic analysis of bacterial composition and for metabolomics analysis. Samples for the analysis of bacterial composition were put in the inhibitEX buffer (from QIAmp fast DNA stool mini-kit) and processed. Fecal bacterial DNA was extracted with QIAmp fast DNA Stool mini-kit (QIAGEN) according to manufacturer’s instructions. The samples for the NMR analysis of metabolites were rapidly frozen in liquid nitrogen and stored at −80 °C until use.

### 16S targeted sequencing

The V2–V3 portion of the 16 S rRNA was amplified, using the primer set F101- R534, with a different IonXpress barcode per sample attached to the reverse primer. PCR reactions were performed using the Kapa Library Amplification Kit (Kapa Biosystems, Massachusetts, USA) and BSA 400 ng/µL, under the following conditions: 5 min at 95 °C, 30 s at 95 °C, 30 s at 59 °C, 45 s at 72 °C, and a final elongation step at 72 °C for 10 min. DNA after normalization was quantified with a Qubit® 2.0 Fluorometer (Invitrogen, Carlsbad, California, USA). The amplicon size was checked on a 2% agarose gel. The subsequent step of PCR purification was carried out using the Mag-Bind® Total Pure NGS beads (OMEGA Bio-Tek, Georgia, USA), retaining fragments >100 bp. Template preparation was performed by the Ion PGM Hi-Q View kit on the Ion OneTouch^TM^ 2 System (Life Technologies, Grand Island, NY, United States) and sequenced using the Ion PGM Hi-Q View sequencing kit (Life Technologies, Grand Island, NY, United States) with the Ion PGM^TM^ System technology. Negative controls, including a no-template control, were processed along with samples as previously suggested^74^.

### Microbiota characterization

Raw FASTQ files were analyzed with DADA2 pipeline v.1.14 for quality check and filtering (sequencing errors, denoising, chimerae detection) on a Workstation Fujitsu Celsius R940 (Fujitsu, Tokyo, Japan). Filtering parameters were as follows: truncLen=0, minLen=100, maxN=0, maxEE=2, truncQ=11, trimLeft=15. All the other parameters in the DADA2 pipeline for single-end IonTorrent were left as default. Raw reads (1792808 in total, on average 89640 per sample) were filtered (562465 in total, on average 28123 per sample) and 1366 Amplicon Sequence Variants (ASV) were found. Sample coverage was computed and resulted to be on average higher than 99% for all samples, thus meaning a suitable normalization procedure for subsequent analyses. Bioinformatic and statistical analyses on recognized ASV were performed with Python v.3.8.2. Each ASV sequence underwent a nucleotide Blast using the National Center for Biotechnology Information (NCBI) Blast software (ncbi-blast-2.3.0) and the latest NCBI 16 S Microbial Database accessed at the end of March 2021 (ftp://ftp.ncbi.nlm.nih.gov/blast/db/). After blasting, the 1366 ASVs were merged into 145 species (thus excluding sub-species or strain differences), and a matrix of their relative abundances was built for subsequent statistical analyses.

### Sample preparation for NMR analysis

#### Fecal water

NMR analysis was conducted on stool samples taken from 8 SE and 10 EE mice. In all, 2 out of 10 samples of the SE group were not analyzed since the fecal material at the time of sampling was too low to ensure a correct quantitative analysis. Each fecal sample was suspended in ice-cold D_2_O–PBS–NaN_3_ buffered solution (1.4 mL), vortexed for 2 min and centrifuged at 11,000 × *g* for 15 min at 4 °C. The supernatant was filtered through a cell strainer (100-μm pore size), centrifuged and filtered again through a sterile syringe filter (0.2-μm pore size). PBS-D_2_O- NaN_3_ (60 μL) containing 20 mM of the internal standard trimethyl silyl propanoic acid (TSP) was added to the supernatants (600 μL) in order to obtain TSP final concentration of 2 mM. Each fecal water sample (final volume of 660 μL) was finally transferred into NMR precision tubes and analyzed by high-resolution NMR spectroscopy.

#### Serum

Twenty-four serum samples (twelve SCFA-treated, twelve controls) were analyzed after five-week treatment. Since the blood sampling method did not guarantee for all samples a reproducible serum volume, which was also below the threshold for the quantitative NMR analysis procedure carried out in this study, two samples were randomly pooled together for each group, in order to obtain six treated samples and six controls. In all, 200 μL of each pooled sample was added to 500 μL of PBS-D_2_O solution and then centrifuged for 15 min at 11,000 × *g*. Finally, 660 μL of supernatant was transferred into NMR precision tubes and analyzed.

### NMR acquirement and processing

All NMR spectra were acquired at 298 K using a JEOL JNM-ECZR spectrometer operating at 14.09 Tesla and 600.17 MHz for ^1^H frequency.

#### Fecal water

Spectra from fecal water were recorded using pulse sequences, including standard ^1^H experiment detection with presaturation (presat) pulse sequence. Spectral width was set to 9.03 KHz (15 ppm) and 128 scans were collected for each spectrum with a presat pulse length of 2.00 s and a relaxation delay of 5.72 s. Spectra were collected with 64 k points for an acquisition time of 5.81 s. The assignment of resonances was conducted by 2D homonuclear Total Correlation Spectroscopy (TOCSY) and heteronuclear Single Quantum Coherence (HSQC) experiments. TOCSY experiments were recorded at 298 K with a spectral width of 15 ppm in both dimensions, using 8 k × 256 data points matrix, repetition time of 3 s and 80 scans with a mixing time of 80 ms. HSQC experiments were acquired with a spectral width of 9.03 KHz (15 ppm) in proton dimension and 30 KHz (200 ppm) in the carbon dimension, using 8 K × 256 data point matrix for the proton and the carbon dimensions, respectively, with a repetition delay of 2 s and 96 scans. In order to confirm the resonances assignment, Human Metabolome Data Base^75^ and in-house laboratory database were consulted. Monodimensional ^1^H NMR spectra were processed and quantified by using the ACD Lab 1D-NMR Manager 12.0 software (Advanced Chemistry Development, Inc., Toronto, ON, Canada), whereas the JEOL Delta v5.3.1 software (JEOL Ltd, Tokyo, Japan) was used to assess 2D-NMR spectra. Fourier transformation of the free induction decays was performed after multiplying by an exponential function with a line broadening of 0.3 Hz. The acquired NMR spectra phased, baseline corrected, and referenced to the chemical shift of the TSP methyl resonance at δ 0.00. Due to the overcrowding of ^1^H-NMR spectra, only those signals that did not overlap with other resonances were considered for integration. The quantification of metabolites was obtained by comparison of the signal’s integral normalized for number of protons with the internal standard TSP integral and then normalized for feces weight. Final data were expressed as µmol/g.

#### Serum

Each spectrum from serum samples was acquired with a Carr-Purcell-Meiboom-Gill (CPMG) presat pulse sequence, with 128 scans collected, 2.72 s relaxation delay, 125 number of loops, 64k spectrum size, a 9.03 kHz spectral width, and processed with a line broadening factor of 0.3 Hz.

The acquired NMR spectra phased, baseline corrected, and referenced to the chemical shift of the lactate quartet resonance at δ 4.11. The quantification of metabolites was obtained by comparison of the signal’s integral normalized for number of protons with the creatine’s integral at 3.95 ppm normalized for number of protons.

### Mice treatments

Three-week old mice were treated for 5 weeks with SCFAs in autoclaved drinking water. The oral mixture of 150 mM sodium formate (Sigma-Aldrich) and 67.5 mM sodium acetate (Sigma-Aldrich) was prepared and changed weekly, assuming an intake of 7 ml per mouse per day. The intake of SCFA solution was calculated by difference with the volume of solution left in the feeding bottle. The stability of SCFA solution kept at RT for 7 days was verified by NMR analysis. The SCFA concentrations used were similar to those used in previous papers^18,60,69,76,77^ and shown not to be toxic in mice. Sodium matched SE groups were used in all the experiments with SCFAs.

### RNA extraction and real-time PCR

Mice were sacrificed by decapitation, the brain were removed, the hemisphere separated, the hippocampi dissected and placed into ice-cold HBSS. The hippocampi were disrupted in a glass-teflon homogenizer as long as the suspension was not clear and passed through a 100 nm nylon cell strainer (Corning). Suspension was centrifuged (1000 × *g*, 10 min, RT) and the pellet lysed in Trizol reagent (Sigma-Aldrich). Total RNA was extracted with Trizol reagent (Sigma-Aldrich). The quality and yield of RNAs were verified using the Nanodrop One System (Thermo Scientific). One μg of total RNA was reverse transcribed in a final reaction volume of 20 μl. Reverse transcription reaction was performed in a thermocycler (MJ Mini Thermal Cycler Biorad) using iScript^TM^ Reverse Transcription Supermix (Biorad) according to the manufacturer’s protocol, under the following conditions: incubation at 25 °C for 5 min, reverse transcription at 46 °C for 20 min, inactivation at 95 °C for 1 min. Real-time PCR (RT-PCR) was carried out in a CFX Real-Time PCR System (Biorad) using SsoFast^TM^ EvaGreen Supermix (Biorad) according to the manufacturer’s instructions. Specific primers pairs, at a final concentration of 500 nM, were used to measure mRNA levels were as follows: glyceraldehyde 3-phosphate dehydrogenase (*gapdh*) F 5′-TTCGCAAAACAAGTTCACCA-3′ and R 5′-TCGTTGTGGTTGTAAATGGAA-3′, brain-derived neurotrophic factor (*bdnf*) F 5′-CCATAAGGACGCGGACTTGTAC-3′ and R 5′-AGACATGTTTGCGGCATCCAGG-3′, nerve growth factor (*ngf*) F: 5′-ACA CTC TGA TCA CTG CGT TTT TG-3′ and R: 5′-CCT TCT GGG ACA TTG CTA TCT GT-3′, epidermal growth factor (*egf*) F 5′-AGC ATA CTC AGC GTC ACA GC-3′ and R 5′-GCA GGA CCG GCA CAA GTC-3′R, insulin-like growth factor (*igf*) F 5′-GTG TGG ACC GAG GGG CTT TTA CTT C-3′ and R 5′-CTT CTG AGT CTT GGG CAT GTC AGT G-3′, vascular endothelial growth factor-a (*vegfa*) F 5′-GAT CAT GCG GAT CAA ACC TC-3′, and R 5′-AAT GCT TTC TCC GCT CTG AA-3′. The PCR protocol consisted of 40 cycles of denaturation at 95 °C for 30 s and annealing/extension at 58 °C for 30 s. Melt curves analyses were performed at the end of every RT-q PCR to confirm formation of a single PCR product. No template controls were added for each target to exclude possible sample contamination. For quantification analysis the comparative threshold Cycle (Ct) method was used. The Ct values from each gene were normalized to the Ct value of *gapdh* in the same RNA samples. Relative quantification was performed using the 2^−ΔΔCt^ method and expressed as fold changes.

### Behavioral tests

Before testing, the mice were placed in the experimental environments for habituation (>10 min). After each test, the apparatus was carefully cleaned with 10% alcohol.

### Open field test

This test is used to assay general locomotor activity levels, anxiety, and willingness to explore. Mice were placed in the corner of an enclosed platform (40 cm × 40 cm × 30 cm). The total distance traveled, movement duration and time spent in the center area (20 cm × 20 cm) were recorded for each mouse, for 10 min with ANY-MAZE software.

### Novel object recognition test

The NORT was performed 24 h after the open field test, in the same platform using discriminated objects of identically sized. This test analyzes the non-spatial working memory function. Two identical objects were presented, and the mouse was allowed to freely explore for 10 min. After 1 h, the mouse was retested for 5 min in the same apparatus, where one of the objects had been replaced by a novel one. Exploration behavior was defined as turning the nose toward the object at a distance ±2 cm or touching it with the nose. The discrimination index was defined as the ratio of time spent exploring the novel object / total amount of time spent exploring both objects. Videos were recorded with ANY-MAZE software.

### Immunofluorescence

Mice were overdosed with chloral hydrate (400 mg/kg, i.p.) and then intra-cardially perfused with PBS and then PFA 4%; brains were then isolated, post-fixed in 4% paraformaldehyde for further 24 h and cryopreserved with 30% sucrose and snap frozen. Immunofluorescence staining procedures were conducted on cryostat sections (10 μm) containing the hippocampal DG starting at −1.46 mm and ending at −2.80 mm from the bregma (for the analysis we collected one section every 240 μm). The whole region of interest was covered by 20× scanslide. The same number of sections for mouse (at least six) was incubated for 1 h in 3% goat serum and 0.3% Triton X-100, in PBS 0.1 M at RT. Sections were then incubated in rabbit anti DCX (Cell Signaling, USA) diluted in 1% goat serum 0.1% Triton X-100, in PBS 0.1 M, for 24 h at 4 °C. After washing in PBS, the sections were incubated in secondary antibody (donkey anti rabbit, Alexafluor, Invitrogen) for 1 h a RT, washed in PBS and then stained with Hoechst (Invitrogen) for 5 min, washed again and mounted on a microscope slide for the analysis of fluorescence (Eclipse, Nikon). DCX^+^ cells in the SGZ and the granule cell layer of the DG were counted exhaustively. In the same slices analyzed for neurogenesis, DG hippocampal area was calculated with MetaMorph software and the counting of DCX^+^ cells were normalized to DG area.

### Slice preparation for electrophysiology

Anesthetized animals were decapitated and the whole brains were rapidly removed from the skull and immersed for 10 min in ice-cold artificial cerebrospinal fluid (ACSF; composition in mM: NaCl 125, KCl 4, CaCl2 2.5, MgSO4 1.5, NaH2PO4 1, NaHCO3 26, glucose 10; 295-300 mOsm), continuously oxygenated with 95% O2 and 5% CO2 to maintain the proper pH (7.4). Transverse 350 µm slices were cut at 4 °C with a vibratome (Thermo Scientific, USA) and then placed in a chamber containing oxygenated ACSF. After their preparation, slices were allowed to recover for at least 1 h at 30 °C.

### Field excitatory post synaptic potential recordings

Slices were transferred to the slice-recording chamber interface (BSC1, Scientific System Design Inc), maintained at 30–32 °C and constantly superfused at the rate of 2.5 ml/min with oxygenated ACSF. At the beginning of each recording, a concentric bipolar stimulating electrode (SNE-100 × 50-mm-long Elektronik–Harvard Apparatus GmbH) was placed in the hippocampus CA1 stratum radiatum for stimulation of Shaffer collateral pathway projection to CA1. Stimuli consisted of 100 µs constant current pulses of variable intensities, applied at 0.05 Hz. A glass micropipette (0.5–1 MΩ) filled with ACSF was placed in the CA1 region, at roughly the same distance from the stimulating electrode, in order to measure orthodromically evoked fEPSP (field excitatory post synaptic potential). Stimulus intensity was adjusted to evoke fEPSP of amplitude ~50% of the maximal amplitude with minimal contamination by a population spike. Evoked responses were monitored online and stable baseline responses were recorded for at least 10 min (variation in amplitude values <10%). Only the slices that showed stable fEPSP amplitudes were included in the experiments. LTP was induced by high-frequency stimulation (HFS, 1 train of stimuli at 100 Hz of 1 s duration). To analyze the time-course of the fEPSP amplitude, the recorded fEPSP was routinely averaged over 1 min (*n* = 3) and normalized to the baseline values. The fEPSP amplitude changes following the LTP induction were calculated with respect to those of the baseline (1 min before induction). n/N refers to the number of slices on the total number of mice analyzed. The paired-pulse ratio (PPR) was measured from responses to two synaptic stimuli at several interstimulus intervals (20-50-70-100-150-200 ms). PPR was calculated as the ratio between the fEPSP amplitude evoked by the second stimulus (A2) and that by the first (A1; A2/A1). fEPSP were recorded and filtered (low pass at 1 kHz) with an Axopatch 200 A amplifier (Axon Instruments, CA) and digitized at 10k Hz with an A/D converter (Digidata 1322 A, Axon Instruments). Data acquisition was stored on a computer using pClamp 9 software and analyzed offline with Clampfit 10 software (both from Axon Instruments).

### Statistics and reproducibility

For metagenomics, data matrices were first normalized then standardized using QuantileTransformer and StandardScaler methods from Sci-Kit learn package v0.20.3. Normalization using the output_distribution = ’normal’ option transforms each variable to a strictly Gaussian-shaped distribution, whilst the standardization results in each normalized variable having a mean of zero and variance of one. These two steps of normalization followed by standardization ensure the proper comparison of variables with different dynamic ranges, such as bacterial relative abundances, or cytokines levels. For microbiota analysis, measurements of α diversity (within sample diversity) such as Richness and Shannon index, were calculated at species level using the SciKit-learn package v.0.4.1. Exploratory analysis of β-diversity (between sample diversity) was calculated using the Bray-Curtis measure of dissimilarity and represented in Principal Coordinate Analyses (PcoA), along with methods to compare groups of multivariate sample units (analysis of similarities - ANOSIM, permutational multivariate analysis of variance - PERMANOVA) to assess significance in data points clustering^78^. ANOSIM and PERMANOVA were automatically calculated after 999 permutations, as implemented in SciKit-learn package v0.4.1. In order to visualize a non-supervised clusterization as PCoA, we implemented with custom scripts (Python v3.8.2) a Hierarchical Clustering Analysis (HCA) with ‘Bray-Curtis’ metrics and ‘complete linkage’ method. We implemented Partial Least Square Discriminant Analysis (PLS-DA) and the subsequent Variable Importance Plot (VIP) as a supervised analysis wherein the VIP values (order of magnitude) are used to identify the most discriminant bacterial species among the cohorts. Bar thickness reports the fold ratio (FR) value of the mean relative abundances for each species among the two cohorts, while an absent border indicates mean relative abundance of zero in the compared cohort. Mann–Whitney U test and P values, without FDR was used for a fixed sample size as previously described^79^ and Kruskal–Wallis tests were employed to assess significance for pairwise or multiple comparisons, respectively, considering a *P* value < 0.05 as significant. We acknowledge that this statistic approach could represent a limitation of our study, and further experiments with higher number of animals will be necessary to further consolidate the results on the effect of housing on microbiota composition. Statistical analyses gathering more than two groups were performed using ANOVA followed by pairwise comparisons with Bonferroni adjustments. Differential enrichment analyses in murine studies were corrected for multiple hypothesis testing using a two-stage Benjamini-Hochberg FDR at 10%.

Pearson matrices for network analysis (metric = Bray-Curtis, method = complete linkage) were generated on normalized and standardized data with in-house scripts (Python v3.8.2) and visualized with Gephi v.0.9.2, as previously reported^80,81^. Bacterial species having a prevalence ≥5% were considered to generate the nodes within the final network, while a significant Pearson correlation coefficient and its related *P* value (after Benjamini–Hochberg FDR at 10%) was employed to obtain eight categories defining edge thickness^82^. A 5-fold cross-validated leave-one-out (5CV-LOO) method was employed by SciKit-learn package v0.4.1 on the subjects in order to have an averaged *P* value for each correlation among two definite variables. Edges were pruned with two-stages FDR 10%. Network analysis was performed on unified datasets^80^ taking care of an optimized visual representation with Gephi v.0.9.2, as proposed by current guidelines^83–87^. Nodes were colored according to the cohort in which species harbored the highest mean relative abundance, after normalization and standardization. The degree value, measuring the in/out number of edges linked to a node, and the betweenness centrality, measuring how often a node appears on the shortest paths between pairs of nodes in a network, were computed with Gephi v.0.9.2. Intra-network communities (Species Interacting Groups, SIGs)^80,88^ were retrieved using the Blondel community detection algorithm^89^ by means of randomized composition and edge weights, with a resolution equal to 1^90^. Species belonging to a definite SIG were defined after 100 iterations of Blondel algorithm, then computing the variation coefficient (CV) for species assignation to a SIG, and grouping the species having the same CV value into a SIG.

For the NMR-based metabolomics, multivariate partial least squares – discriminant analysis (PLS-DA) was performed using in-house routines running under MATLAB R2015b environment (The MathWorks, Natick, MA, USA), and a double cross validation procedure was employed to verify the statistical reliability of the PLS-DA model^91^. Variable importance in projection (VIP) indices were used to assess which variable contributed the most to the classification model. Univariate statistical analysis was carried out by SigmaPlot 14.0 (Systat Software, Inc, San Jose, CA, USA) and Mann–Whitney *U* test was run to determine significant differences for considered metabolites. A *p*-value < 0.05 was considered statistically significant.

For electrophysiological recordings, data are shown as the mean ± SEM. Statistical significance was assessed by Student’s *t* test or ANOVA analysis and Dunn’s, Student-Neuman-Keuls’s, Dunnet’s test and Fisher’s LSD was used as a post-hoc test as indicated. The exact *p*-values are indicated in the text where available and the multiplicity-adjusted *p*-values are indicated in the corresponding figures (*p < 0.05, **p < 0.01, ***p < 0.001) corresponding figures. Statistical analyses comprising calculation of degrees of freedom were done GraphPad Prism 8.0.1 software.

### Reporting summary

Further information on research design is available in the Nature Research Reporting Summary linked to this article.

## Supplementary information

Description of Additional Supplementary Files

Supplementary Information

Supplementary Data 1

Supplementary Data 2

Supplementary Data 3

Supplementary Data 4

Supplementary Data 5

Supplementary Data 6

Reporting summary

## Data Availability

Raw fastq files were submitted to NCBI Sequence Read Archive (SRA) portal under the Bioproject PRJNA722696, submission SUB9477301. The source data underlying Figs. 1a, d, f, 2b, c, 3, and 4 are present in Supplementary Data 5, 3, and 6 respectively. Any other relevant data are available upon reasonable request.
