# Supplementary material for: Short-chain fatty acids promote the effect of environmental signals on the gut microbiome and metabolome in mice
Source: Commun Biol. 2022 May 31;5:517. doi: 10.1038/s42003-022-03468-9 (PMC9156677; doi:10.1038/s42003-022-03468-9)
Supplement: Supplementary file 1 — Description of Additional Supplementary Files [file 42003_2022_3468_MOESM1_ESM.pdf]

Description of Additional Supplementary Files

File Name: Supplementary Data 1

Description: Pairwise analysis between SE and EE species abundance

File Name: Supplementary Data 2

Description: Table reporting the level of assignment for SE and EE fecal metabolites.

File Name: Supplementary Data 3

Description: Abundances of fecal metabolites in SE and EE, source data for figure 2b and 2c

File Name: Supplementary Data 4

Description: Source data for supplementary figure 5

File Name: Supplementary Data 5

Description: Source data for figure 1a, 1d and 1f

File Name: Supplementary Data 6

Description: Source data for figures 3 and 4
