# Supplementary material for: Short-chain fatty acids promote the effect of environmental signals on the gut microbiome and metabolome in mice
Source: Commun Biol. 2022 May 31;5:517. doi: 10.1038/s42003-022-03468-9 (PMC9156677; doi:10.1038/s42003-022-03468-9)
Supplement: Supplementary file 2 — Supplementary Information [file 42003_2022_3468_MOESM2_ESM.pdf]

Supplementary Figures and Tables

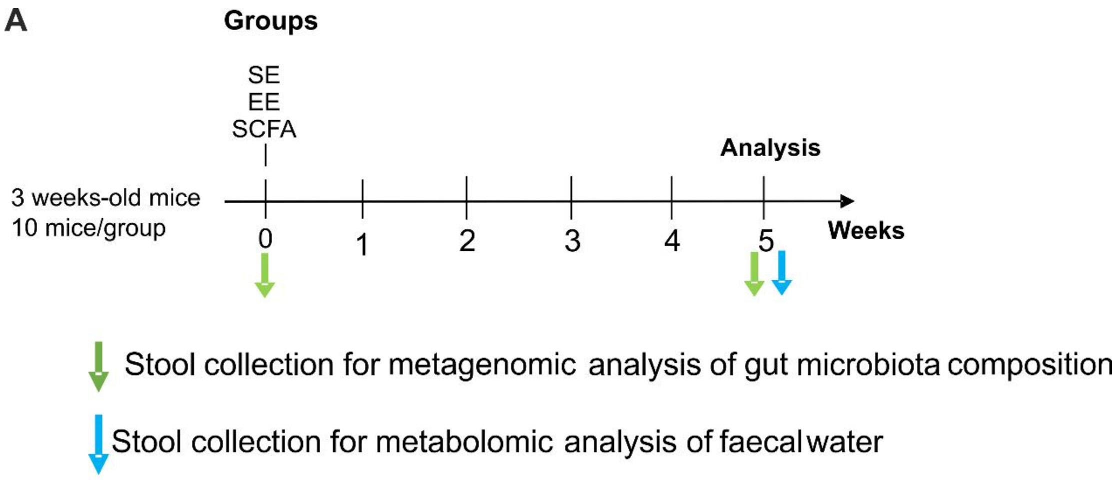

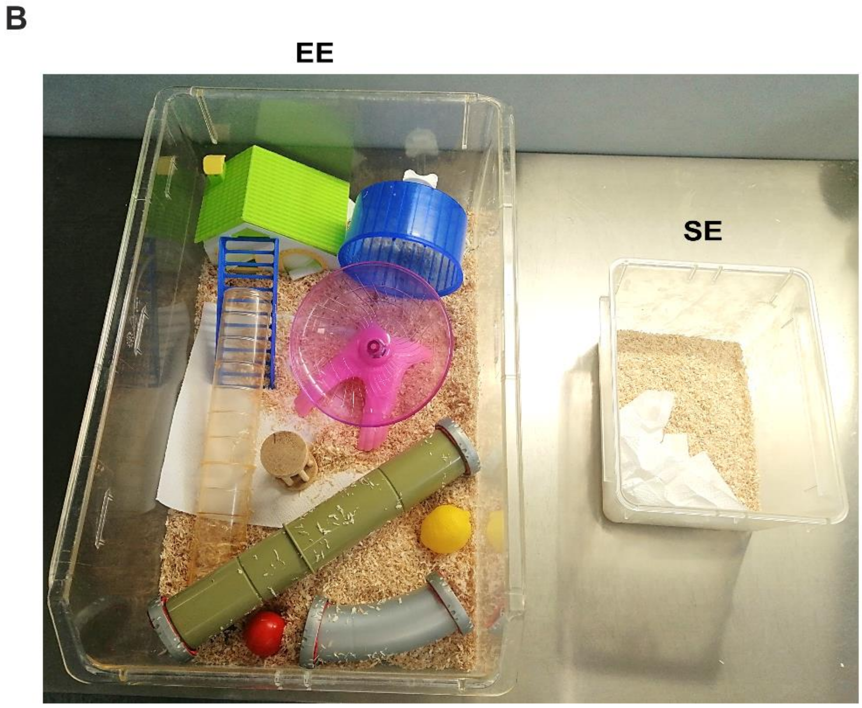

Supplementary Figure 1: a. Experimental plan showing animal groups (at least ten mice), age, and timing of stool collection and analysis. **b**. Picture showing the different housing conditions: Environmental Enrichment (EE) on the left and Standard Environment (SE) on the right.

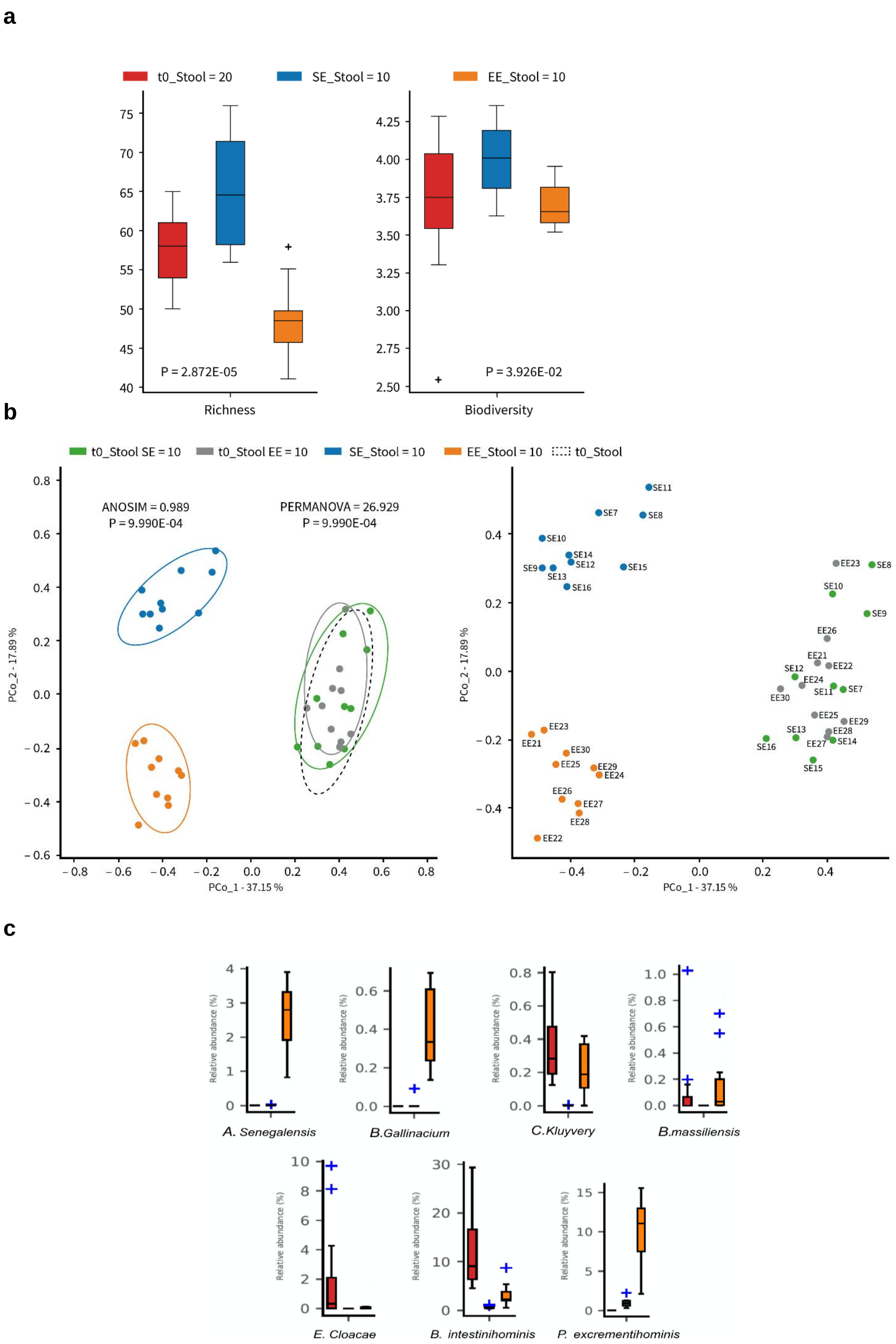
a
t0_Stool = 20
SE_Stool = 10
EE_Stool = 10
P = 2.872E-05
Richness
P = 3.926E-02
Biodiversity
b
t0_Stool SE = 10
t0_Stool EE = 10
SE_Stool = 10
EE_Stool = 10
t0_Stool
ANOSIM = 0.989
P = 9.990E-04
PERMANOVA = 26.929
P = 9.990E-04
PCo_2 - 17.89 %
PCo_1 - 37.15 %
c
Relative abundance (%)
A. Senegalensis
B.Gallinacium
C.Kluyvery
B.massiliensis
E. Cloacae
B. intestinihominis
P. excrementihominis

Supplementary Figure 2: a. Alpha-diversity analysis for microbiota richness (number of bacterial species) and biodiversity (Shannon metric) at time zero (t0) and after 5 weeks in EE or in SE. Beta-diversity analysis **(b, left)** at time zero (t0) and after 5 weeks in EE or in SE. **Right**, the same analysis showing the mouse number in t0 group (baseline, 20 mice named *SE 7-16, EE 21-30* for their future destiny), in EE group (10 mice named *EE 21-30)* and in SE group (10 mice named *SE 7-16)*. **c.** Analysis of 7 selected species (belonging to EE-related SIG1) depicts significant differences in terms of relative abundance when matched with t0. P value from multiple comparison analysis among t0 and SE and EE cohorts are the follow: *A. senegalensis* P=1,821-07 *EE vs t0*; *B. gallinacium* P=2.264E-07 *EE vs t0*; *C. kluyveri* P= 2.350E-05 SE vs t0 and P= 0.031 *EE vs t0*; *B. massiliensis* P= 0.015 *SE vs t0*; *E. cloacae* P= 0.0007 *SE vs t0* and P= 0,029 *EE vs t0*; *B. intestinihominis* P= 0.000012 *SE vs t0* and P= 0,0001 *EE vs t0*; *P. excrementihominis* P=5.94E-07 *SE vs t0* and P=2.27E-07 *EE vs t0*. The boxplots show the minimum, 25th percentile, median, 75th percentile, and maximum values. Error bars = SEM

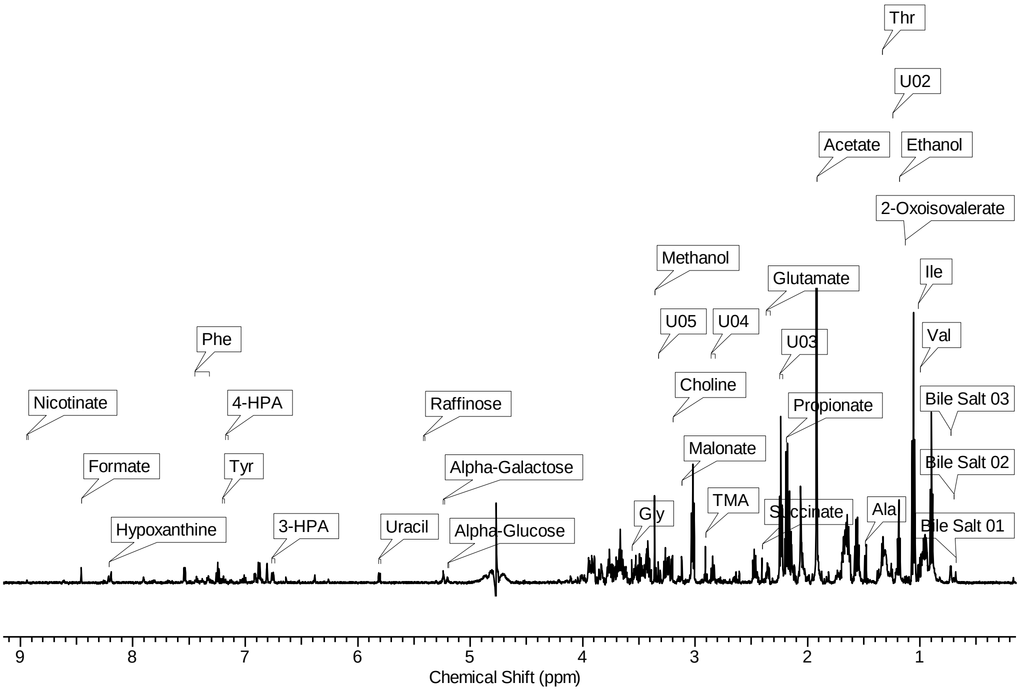

Supplementary Figure 3: Representative ^{1}H NMR spectrum of fecal water. The assignment of the resonances is reported in Table S1

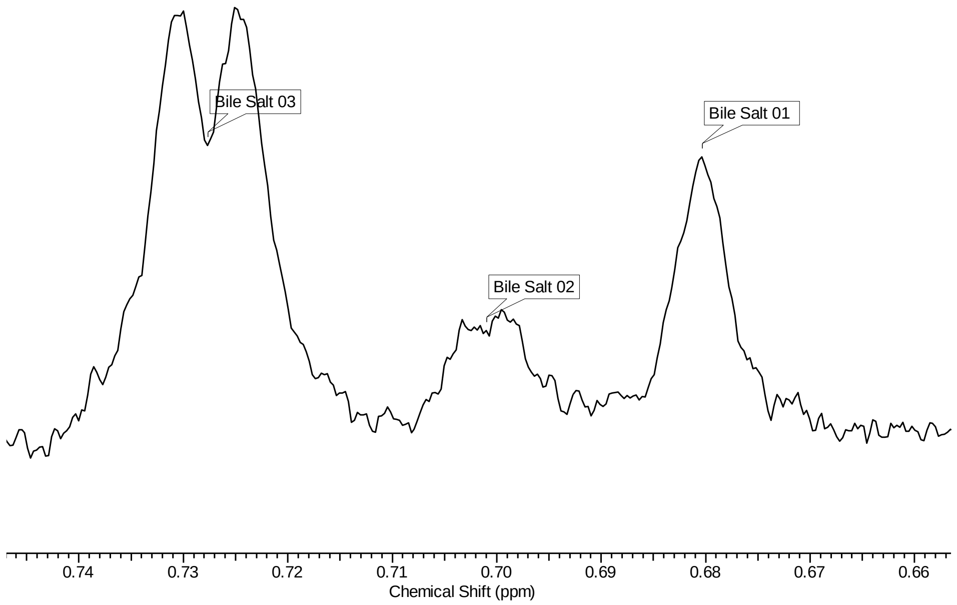

Supplementary Figure 4: 1H NMR spectrum of C-18 bile salts moieties

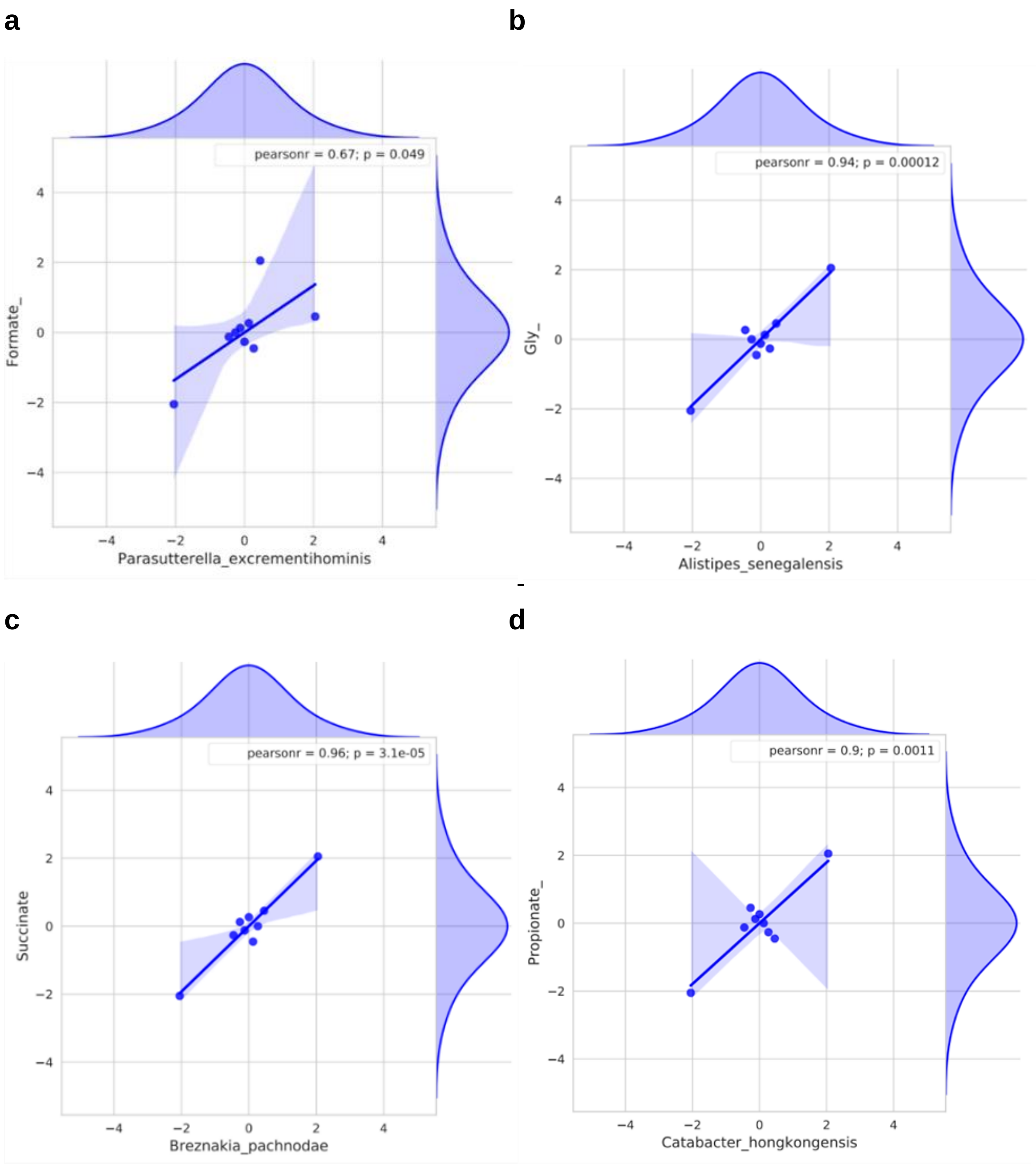

Supplementary Figure 5: Raw data (each dot represents 1 mouse) of the Pearson correlations between *Parasutterella. excrementihominis* and the formate **(a),** *Alistipes senegalensis* and glycine **(b),** *Breznakia pachnodae* and succinate **(c)** and *Catabater hongkongensis* and propionate **(d).**

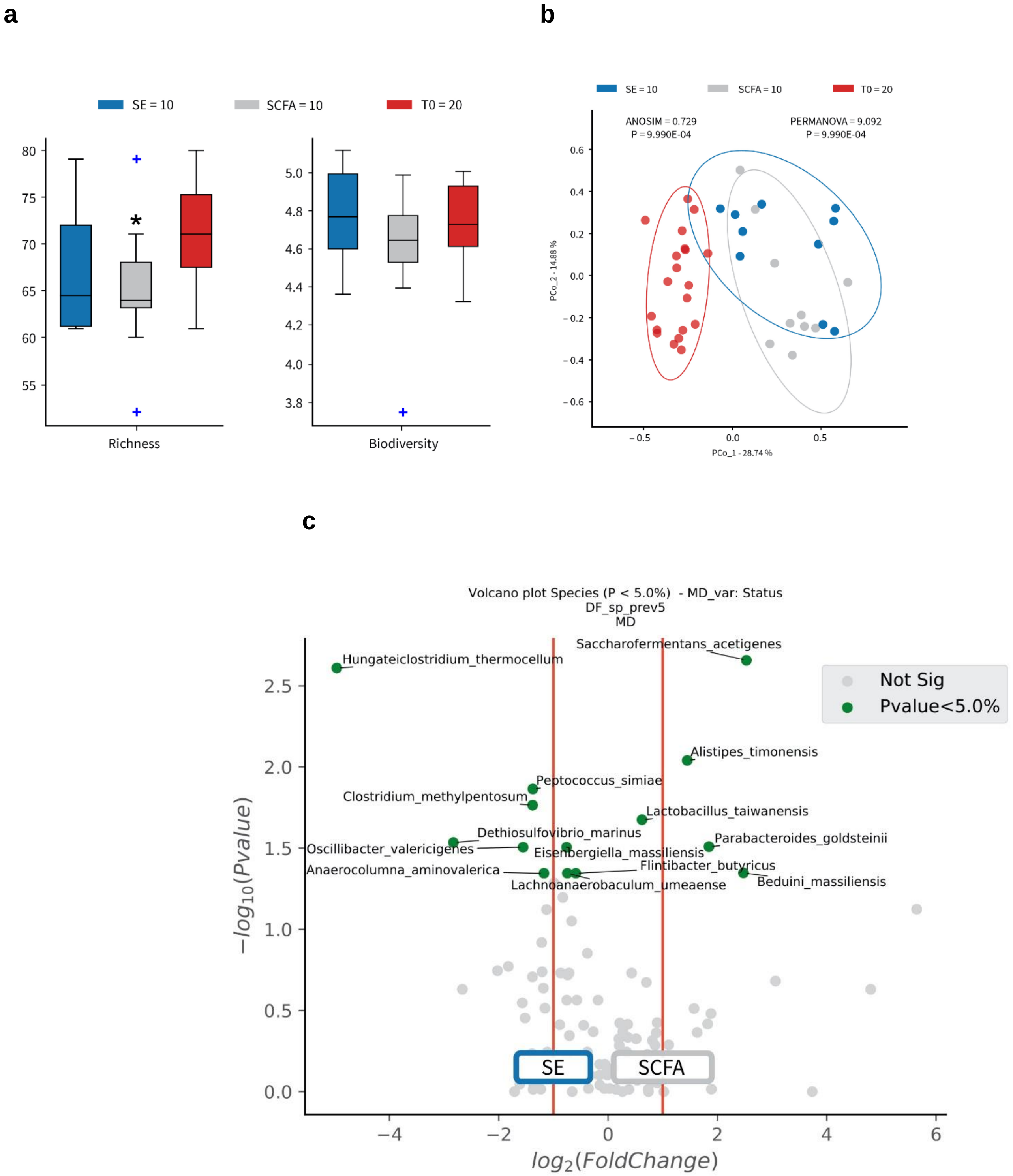

Supplementary Figure 6: Alfa-diversity **(a)** shows differences (Kruskal-Wallis test) in richness (number of bacterial species) but not in biodiversity (Shannon metric) among baseline (T0, red, n=20) and short chain fatty acid (SCFA, grey, n=10 p= 0.014). **b.** Beta-diversity depicts differences (Bray-Curtis distance) among groups in terms of microbiota species composition. **c.** Volcano Plot shows discriminant species of fecal microbiome between SE and SCFA groups (in green species significantly different for their relative abundances following Mann-Whitney U test, without two-stages FDR at 10%). The boxplots show the minimum, 25th percentile, median, 75th percentile, and maximum values. Error bars = SEM

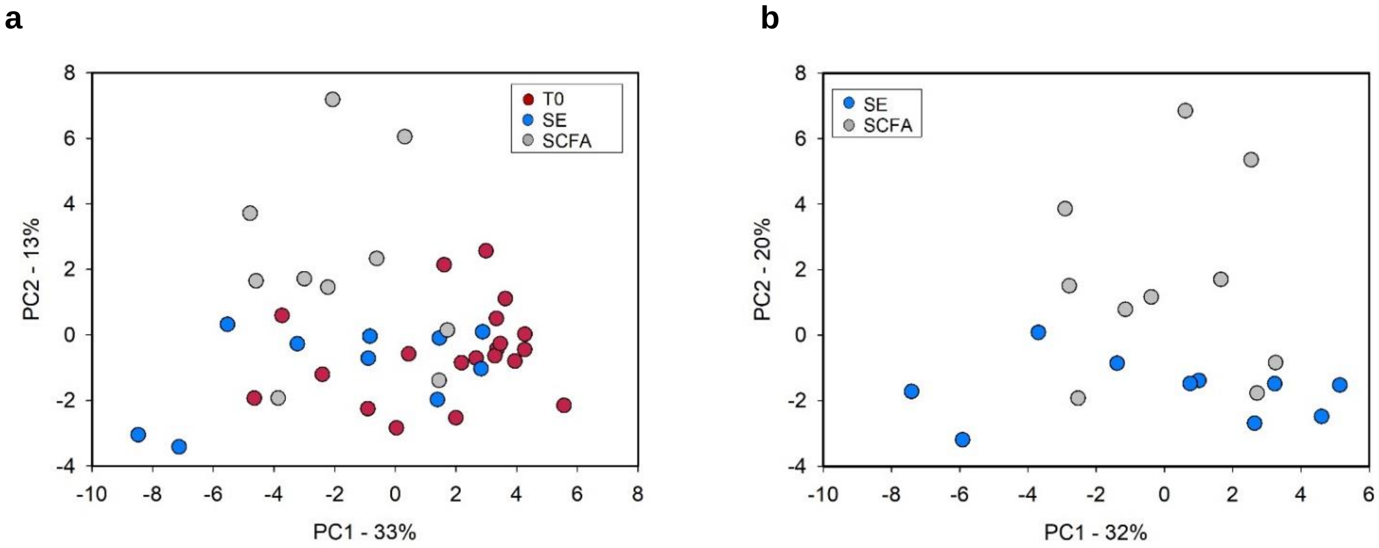

Supplementary Figure 7: PCA score plot from **a)** time zero (T0, red, n=20), standard environment (SE, blue, n=10) and short chain fatty acid (SCFA, grey, n=10) mice data set and **b)** SE and SCFA treated mice data set. Here is shown an apparent separation between the treated and not treated samples along the PC2 (20% of the total explained variance).

Supplementary Table 1: ^{1}H NMR assignment of mice's fecal waters. The level of assignment has been reported according to Salek M. R. et al [1].

The Supplementary Table 1 is reported as Supplementary Data File 2.

Supplementary References

1. Salek, R. M., Steinbeck, C., Viant, M. R., Goodacre, R. & Dunn, W. B. The role of reporting standards for metabolite annotation and identification in metabolomic studies. *GigaScience* **2**, 2047-217X-2–13 (2013).
