# Supplementary material for: Short-chain fatty acids promote the effect of environmental signals on the gut microbiome and metabolome in mice
Source: Commun Biol. 2022 May 31;5:517. doi: 10.1038/s42003-022-03468-9 (PMC9156677; doi:10.1038/s42003-022-03468-9)
Supplement: Supplementary file 9 — Reporting summary [file 42003_2022_3468_MOESM9_ESM.pdf]

nature research

Corresponding author(s): Cristina Limatola

Last updated by author(s): Mar 30, 2022

Reporting Summary

Nature Research wishes to improve the reproducibility of the work that we publish. This form provides structure for consistency and transparency in reporting. For further information on Nature Research policies, see our Editorial Policies and the Editorial Policy Checklist.

Statistics

For all statistical analyses, confirm that the following items are present in the figure legend, table legend, main text, or Methods section.

n/a	Confirmed	
☐	☒	The exact sample size (*n*) for each experimental group/condition, given as a discrete number and unit of measurement
☐	☒	A statement on whether measurements were taken from distinct samples or whether the same sample was measured repeatedly
☐	☒	The statistical test(s) used AND whether they are one- or two-sided<br>*Only common tests should be described solely by name; describe more complex techniques in the Methods section.*
☐	☒	A description of all covariates tested
☐	☒	A description of any assumptions or corrections, such as tests of normality and adjustment for multiple comparisons
☐	☒	A full description of the statistical parameters including central tendency (e.g. means) or other basic estimates (e.g. regression coefficient) AND variation (e.g. standard deviation) or associated estimates of uncertainty (e.g. confidence intervals)
☐	☒	For null hypothesis testing, the test statistic (e.g. *F*, *t*, *r*) with confidence intervals, effect sizes, degrees of freedom and *P* value noted<br>*Give P values as exact values whenever suitable.*
☐	☒	For Bayesian analysis, information on the choice of priors and Markov chain Monte Carlo settings
☐	☒	For hierarchical and complex designs, identification of the appropriate level for tests and full reporting of outcomes
☐	☒	Estimates of effect sizes (e.g. Cohen's *d*, Pearson's *r*), indicating how they were calculated

Our web collection on statistics for biologists contains articles on many of the points above.

Software and code

Policy information about availability of computer code

Data collection	*Provide a description of all commercial, open source and custom code used to collect the data in this study, specifying the version used OR state that no software was used.*
Data analysis	Statistical analyses were done with the following software: GraphPad Prism8.0.1, SigmaPlot 14.0, SciKit-learn package v.0.4.1., Python v3 .8.2, MATLAB R2015b.

For manuscripts utilizing custom algorithms or software that are central to the research but not yet described in published literature, software must be made available to editors and reviewers. We strongly encourage code deposition in a community repository (e.g. GitHub). See the Nature Research guidelines for submitting code & software for further information.

Data

Policy information about availability of data

All manuscripts must include a data availability statement. This statement should provide the following information, where applicable:

- Accession codes, unique identifiers, or web links for publicly available datasets
- A list of figures that have associated raw data
- A description of any restrictions on data availability

Raw fastq files were submitted to NCBI Sequence Read Archive (SRA) portal under the Bioproject PRJNA722696, submission SUB9477301.

Field-specific reporting

Please select the one below that is the best fit for your research. If you are not sure, read the appropriate sections before making your selection.

☒ Life sciences ☐ Behavioural & social sciences ☐ Ecological, evolutionary & environmental sciences

For a reference copy of the document with all sections, see nature.com/documents/nr-reporting-summary-flat.pdf

Life sciences study design

All studies must disclose on these points even when the disclosure is negative.

Sample size	The sample size (n) was chosen differently for experiments of animal survival, marker expression, etc.. considering the following relation: n≥2sigma(Zalpha/D)2<br>where sigma is substituted by an extimate of variance (s2); alpha is at 0.05 (and Zalpha=-2) and D is the difference among treatments.
Data exclusions	We did not exclude animals from the study.
Replication	All attemps to replicate the results were successful
Randomization	Animals were randomly allocated in the different condition groups (standard or environmental enrichment, control and SCFA)
Blinding	The investigators performing behavioral test, RT-PCR, immunofluorescence, metagenomic, or metabolomic analyses always received the samples from a third laboratory member, not involved in that specific experiment.

Reporting for specific materials, systems and methods

We require information from authors about some types of materials, experimental systems and methods used in many studies. Here, indicate whether each material, system or method listed is relevant to your study. If you are not sure if a list item applies to your research, read the appropriate section before selecting a response.

Materials & experimental systems

n/a	Involved in the study	
☐	☒	Antibodies
☒	☐	Eukaryotic cell lines
☒	☐	Palaeontology and archaeology
☐	☒	Animals and other organisms
☒	☐	Human research participants
☒	☐	Clinical data
☒	☐	Dual use research of concern

Methods

n/a	Involved in the study	
☒	☐	ChIP-seq
☒	☐	Flow cytometry
☒	☐	MRI-based neuroimaging

Antibodies

Antibodies used	rabbit anti-DCX (# 4604s) Cell Signaling, USA
Validation	This antibody is higly used to stain neural progenitors, it obtained 136 citations (most of them using the Ab for immunofluorescence) and the manifacturer certifies Immunofluorescence (Frozen) as approved application.

Animals and other organisms

Policy information about studies involving animals; ARRIVE guidelines recommended for reporting animal research

Laboratory animals	For experiments with animals, we used adult male C57BL/6N mice
Wild animals	The study did not involve wild animals
Field-collected samples	Mice were housed in standard or larger cages at a constant temperature (22 ± 1◦C) and relative humidity (50%), with a 12:12 h light:dark cycle (light on 07.00–19.00 h). Food and water were available ad libitum. Microbiological analyses were routinely (each 3–4 months) in our conventional animal facility.
Ethics oversight	Experiments described in the present work were approved by the Italian Ministry of Health (authorization n. 775/2020-PR) in accordance with the guidelines on the ethical use of animals from the European Community Council Directive of September 22, 2010 (2010/63/EU), and from the Italian D.Lgs 26/2014

Note that full information on the approval of the study protocol must also be provided in the manuscript.
